# The efficacy and safety of furazolidone-bismuth quadruple therapy for *Helicobacter pylori* eradication with or without probiotic supplementation

**Published:** 2022

**Authors:** Nafeh Noorbakhsh, Shahriar Nikpour, Mohammad Salehi

**Affiliations:** 1 *Department of Internal* *Medicine, Shahid Beheshti University of Medical Sciences, Tehran, Iran*; 2 *Department of Internal Medicine, Shahid Beheshti University of Medical* *Sciences, Tehran, Iran*

**Keywords:** H.pylori, Furazolidone, Bismuth, Eradication rate, Probiotic

## Abstract

**Aim:**

In this clinical trial we use furazolidone-bismuth quadruple therapy with or without probiotics for *H.pylori* eradication.

**Background:**

Increasing rates of eradication failure in *H.pylori* infection mainly due to antibiotic resistance has led to search for alternative regimens such as using novel antibiotics and/or using probiotic supplementation as conjunctive to the standard eradication regimens.

**Methods:**

This double blind clinical trial was performed in gastrointestinal clinic of Loghman Hakim University Hospital, Tehran, Iran. Patients with a positive pathology test for *H.pylori* were enrolled to the study and received a 14 day course of furazolidone 100 mg q.i.d, bismuth 240 mg b.i.d, amoxicillin 1000 mg b.i.d, pantoprazole 40 mg b.i.d plus either probiotic (Familact) b.i.d or placebo b.i.d. Adverse effects and adherence to therapy were evaluated at the end of the treatment course. Eradication was established by *H.pylori* fecal antigen test.

**Results:**

A total of 200 patients entered the study and were randomly assigned to two groups of placebo and probiotic. There was no significant difference regarding age or gender between placebo and probiotic groups. Adherence to therapy was higher than 90% in total and not significantly different between placebo and probiotic groups. Total eradication rate was 80.5% (n=161). Eradication rate was 84% in probiotic group vs 77% in placebo group (P=0.2). Total rate of adverse effects was 30% in probiotic group vs 62% in placebo group. The most common adverse effects were abdominal pain (15% in probiotic group vs 28% in placebo group, P=0.03) followed by diarrhea (5% in probiotic group vs 12% in placebo group, P=0.1).

**Conclusion:**

According to our results, adding probiotic to furazolidone-bismuth quadruple therapy did no increase the eradication rate significantly. However, adverse effects particularly abdominal pain was lower in the probiotic group when compared with placebo.

## Introduction

As a very common bacterial infection with a prevalence as high as 80% in developing countries such as Iran, Helicobacter pylori (*H. pylori*) is considered a serious public health issue (1). This pathogen is known to be closely associated with the development of several gastrointestinal disorders including chronic gastritis, peptic ulcer disease, gastric cancer, and mucosa-associated lymphoid tissue (MALT) lymphoma (2-5). Being associated with precancerous conditions, the eradication of *H. pylori* infection is recommended by several international guidelines (6-10).

Failure to eradicate *H. pylori* infection has been reportedly increasing during the last decades with failure rates exceeding 20% in some countries (11). The most important factor responsible for increased failure rates is widespread antibiotic resistance resulting from overuse and misuse of antibiotics (12, 13). Several studies from Iran have demonstrated increasing rates of antibiotic resistance in *H. pylori* infection (14-17). To overcome this issue, several alternative strategies have been suggested, such as using novel antibiotics and/or adding probiotics to the standard eradication regimens (17-20). 

Probiotics are living microorganisms from bacteria or yeast strains that have shown to be effective in varied gastrointestinal conditions including their effect on *H. pylori* infection (21, 22). According to the literature, the effects of probiotics on *H. pylori* infection are associated with both direct anti-bacterial action as well as a reduction in antibiotic-related adverse effects (23, 24). Despite this beneficial line of evidence for probiotics, there is no consensus on their clinical application in *H. pylori* eradication regimens in the guidelines (25-28). 

In the current study, we compare the effect of furazolidone-bismuth quadruple therapy with or without probiotics on *H. pylori* eradication rates, patient adherence rates, and adverse effects. 

## Methods

**Figure 1:**
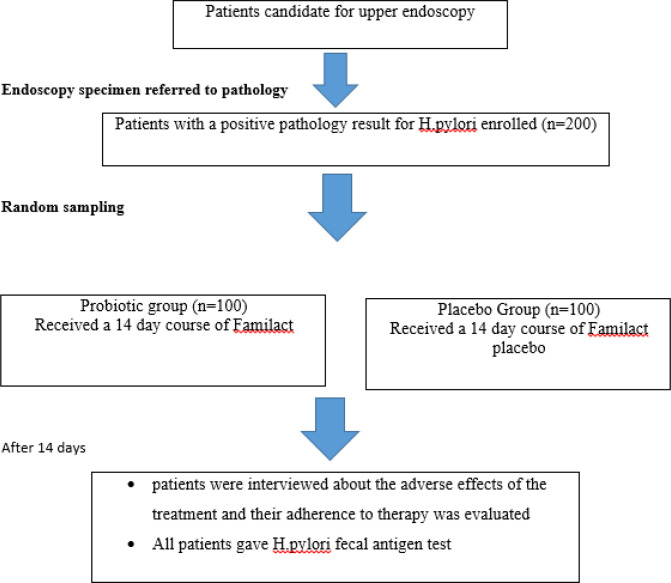
Study methods flow chart

This double blind randomized clinical trial was performed from September 2019 to September 2020 in Loghman Hakim University Hospital, Tehran, Iran. The study protocol was approved by the Ethics Committee of Shahid Beheshti Medical University with an RCT registry code of IR.SBMU.MSP.REC.1397.682 (https://ethics.research.ac.ir). 

Patients older than eighteen years of age who referred to the gastrointestinal clinic of Loghman Hakim University Hospital with chronic dyspepsia and were indicated for upper endoscopy were enrolled in the study. Patients with a history of previous *H. pylori* eradication treatment, antibiotic use within the prior four weeks, and those with severe liver dysfunction, end stage renal disease, severe pulmonary dysfunction, or chronic heart failure, pregnant women, patients with acute peptic ulcer bleeding, and those with G6PD deficiency were excluded. 

All patients underwent upper endoscopy in which several biopsies were taken and sent for pathology testing. Patients with a positive pathology result for *H. pylori* were referred to the clinic. After they were given a thorough explanation about the importance of eradication therapy and the purpose of the study, informed written consent was obtained from those participating in the study. A full medical history was taken, and patients were then randomly assigned to two groups. Each patient received a package containing a 14-day course of furazolidone 100 mg q.i.d., bismuth 240 mg b.i.d., amoxicillin 1000 mg b.i.d., pantoprazole 40 mg b.i.d., plus either Familact b.i.d. or placebo b.i.d. Familact (Zist Takhmir Pharmaceutical Company) is a synbiotic formulation (probiotic plus prebiotic) containing 9 strains of probiotics (i.e. lactobacillus acidophilus, lactobacillus casei, lactobacillus rhamnosus, lactobacillus salivarius, lactobacillus ruteri, bifidobacterium lactis, bifidobacterium langum, bifidobacterium bifidum, and fructooligosaccharide as prebiotic) available as enteric capsules.

**Table 1:** Results of age distribution in study groups

Study Group				
Probiotic group (n=100)			Placebo Group (n=100)	
Gender	Mean age (years)	St. deviation	Mean age (years)	St. deviation
Male	48.24 (n=41)	13.12	47.83 (n=34)	13.42
Female	47.23 (n=59)	14.02	46.94 (n=66(	13.32

**Table 2:** Compares the major adverse effects reported by patients in the two groups

				Group				
		Total		Probiotic		Placebo		P-value
Abdominal painh	No	157(78.5%)		85 (85%)		72 (72%)		0.038
	Yes	43 (21.5%)		15 (15%)		28 (28%)		
Nausea	No	173 (86.5%)		90 (90%)		83 (83%)		0.214
	Yes	27 (13.5%)		10 (10%)		17 (17%)		
Vommiting	No	196 (98)		100 (100%)		96 (96%)		0.121
	Yes	4 (2)		0		4 (4%)		
Diarrhea	No	183 (91.5%)		95 (95%)		88 (88%)		0.126
	Yes	17 (8.5%)		5 (5%)		12 (12%)		
Palpitation	No	199 (99.5)		100 (100%)		99 (99%)		0.999
	Yes	1 (0.5%)		0		1 (1%)		

All patients were scheduled for a second clinic visit within 14 days and were asked to bring their medication package with them. At this visit, all patients were interviewed about the adverse effects of the treatment, and their adherence to therapy was evaluated by counting the percentage of the medications taken. A patient was considered loyal to therapy if they had consumed equal to or higher than 90% of the medication. All patients gave an *H. pylori* fecal antigen test at the end of the 14-day treatment course to establish *H. pylori* eradication. Finally, "per protocol analysis” was performed. 

## Results

A total of 200 patients with positive gastric pathology results for *H. pylori* entered the study and were equally and randomly assigned to either the placebo or the probiotic group. Mean patient age was 47.413.4 years (age range from 21 to 78 years), and 63% (n=126) of participants were female. There was no significant difference regarding age or gender between the placebo and probiotic groups. Adherence to therapy (consumption of more than 90% of the medications) was higher than 90% in total and not significantly different between the placebo and probiotic groups. Results by age are summarized in Table 1.

Total eradication rate, defined as a negative fecal *H. pylori* antigen test, was 80.5% (n=161). (Fecal *H. pylori* antigen test had sensitivity and specificity of 97% and 95%, respectively.) Eradication rate was 84% in the probiotic group and 77% in the placebo group (*p*=0.2). 

Total rate of adverse effects (including abdominal pain, nausea, vomiting, diarrhea, and palpitation) reported by patients was 30% in the probiotic group compared with 62% in the placebo group, but the difference did not reach statistical significance. The most commonly reported adverse effects were abdominal pain (15% in probiotic group, 28% in placebo group; *p*=0.03) followed by diarrhea (5% in probiotic group, 12% in placebo group; *p*=0.1). Table 2 compares the major adverse effects reported by patients in the two groups.

## Discussion

Considering the high prevalence of *H. pylori* infection and its consequences as well as the increasing rates of antimicrobial resistance in Iran, the reduction in *H. pylori* eradication rates has led to the search for alternative eradication regimens (29-33).

One of these alternative treatments has been the use of novel antibiotics such as furazolidone. Furazolidone is a broad spectrum nitrofuran antimicrobial which was already proven to be effective against *H. pylori,* but its use was limited because of side effects and drug interactions which led to decreased patient adherence (34, 35). Because this antibiotic is locally available, safe, and cost effective in Iran, several studies have focused on determining the optimal effective dosage of it in combination with bismuth in quadruple therapies (36-38). According to a review article by Mohammadi et al. (39), the dosage of 100 mg furazolidone q.i.d. (high dose) in the form of a 14-day bismuth-containing quadruple therapy showed the best cure rate with acceptable side effects. 

Accordingly, in the current clinical trial, furazolidone-bismuth quadruple therapy was used for eradication of *H. pylori* and consisted of a 14-day period of furazolidone 100 mg q.i.d., bismuth 240 mg b.i.d., amoxicillin 1000 mg b.i.d., and pantoprazole 40 mg b.i.d. for all patients. The total cure rate was found to be 80.5% with higher than 90% adherence to therapy among patients.

Another alternative suggested for overcoming eradication failure resulting from antimicrobial resistance is the use of probiotic supplementation conjunctive to other standard eradication regimens. Probiotics are bacterial and in some cases yeast species that, when used in particular amounts, can have beneficial health effects in preventing and treating various medical conditions (21, 22, 40). Recent studies have shown a role for probiotic supplementation in the treatment of *H. pylori* infection. While each probiotic has a specific mechanism of action against *H. pylori*, some general mechanisms by which probiotics can contribute to *H. pylori* eradication include strengthening the mucosal barrier, competing for adhesion, secreting antimicrobial compounds, and immunomedulatory actions (41-46). 

In addition to their direct beneficial effects against *H. pylori*, probiotics have also been shown to be effective in improving eradication rates by increasing patient compliance because of the reduced adverse effects of antibiotic therapy. This effect is more prominent when using high doses of antibiotics, as in the current study (47-50). Consistently, adding probiotics to furazolidone bismuth quadruple therapy in the current study led to a reduction in adverse effects, particularly abdominal pain. 

Despite the evidence supporting the beneficial role of probiotics as adjunctive in *H. pylori* eradication regimens, it is not enough to reach a conclusion on their routine clinical application in anti-*H. pylori* therapies. Several studies have shown that probiotics had no effect on eradication rates, although they could reduce the adverse effects (mainly diarrhea) of antibiotics (51-53). In their recent study, Zagari et al. showed that probiotic supplementation did not improve the efficacy or tolerability of the treatment, regardless of the species of the probiotic (54). Thus, there is still no consensus on the use of probiotics in *H. pylori* treatment guidelines. In the present clinical trial, we compared use of probiotics against a placebo added to 14-day furazolidone bismuth quadruple therapy. The results indicated a reduction in adverse effects and an increase in the eradication rate, although none of them reached statistical significance. While some guidelines suggest adding probiotics to standard anti-*H. pylori* treatments mainly because of their role in decreasing the adverse effects of antibiotics, others do not suggest their routine use, as the supporting studies lack quality (26-28). Moreover, the optimal dosage, duration, and role of specific strains of probiotics are yet to be determined, although there is some evidence that a combination of several strains might be preferred (55). The probiotics used in the present study, Familact (Zist Takhmir Pharmaceutical Company), are available locally in a cost-effective enteric capsule with a synbiotic formulation (probiotic plus prebiotic), and its addition to the standard therapy resulted in a significant decrease in abdominal pain as the most commonly reported adverse effect in this study. In addition, all other adverse effects as well as the total rate of adverse effects were reduced in the probiotic group when compared to the placebo group. The total eradication rate in the current study was 80.5%; although it was higher in the probiotic group, the difference was not statistically significant. 

Further large-scale clinical trials are required to determine the definite role and optimal dosage, type, and duration of probiotic supplementation in *H. pylori* eradication regimens.

## Conflict of interests

The authors declare that they have no conflict of interest.
